# Divergent transcriptomic pathways underlie sex-biased cognitive rescue by developmental GSK3B inhibition in a mouse model of 22q11.2 deletion syndrome

**DOI:** 10.1038/s41398-026-04108-0

**Published:** 2026-06-11

**Authors:** Johannes Passecker, Chia-Yuan Chang, Aleksandra Dagunts, Chloe M. Aloimonos, Lukas Leitner, Arsenii Petryk, Florence F. Wagner, Maxym V. Myroshnychenko, David A. Kupferschmidt, Joseph A. Gogos, Joshua A. Gordon

**Affiliations:** 1 Integrative Neuroscience Section, National Institute of Neurological Disorders and Stroke, Bethesda, MD 20892 USA; 2 Institute of Systems Neuroscience, Medical University of Innsbruck, Innsbruck, Austria; 3 Mortimer B. Zuckerman Mind Brain and Behavior Institute, Columbia University, New York, NY 10027 USA; 4 Department of Psychiatry, Vagelos College of Physicians & Surgeons, Columbia University, New York, NY 10032 USA; 5 The Broad Institute of MIT and Harvard Center for the Development of Therapeutics, Cambridge, MA 02142 USA; 6 Departments of Physiology and Cellular Biophysics, Vagelos College of Physicians & Surgeons, Columbia University, New York, NY 10032 USA; 7 Department of Neuroscience, Vagelos College of Physicians & Surgeons, Columbia University, New York, NY 10032 USA; 8 Stavros Niarchos Foundation Center for Precision Psychiatry and Mental Health, Columbia University, New York, NY 10032 USA; 9 New York State Psychiatric Institute, New York, NY 10032 USA

**Keywords:** Learning and memory, Physiology, Genomics

## Abstract

Neuropsychiatric disorders such as schizophrenia frequently exhibit marked sex differences in onset, clinical features, and treatment response. However, the molecular and developmental bases of these differences remain poorly defined. Here, we report a sex-dependent effect of developmental, paralog-selective GSK3B inhibition on working memory (WM) in the *Df(16)A*^*+/−*^ mouse model of 22q11.2 deletion syndrome (22q11.2DS), a genetic condition conferring high risk for schizophrenia. Pharmacological inhibition of GSK3B with BRD3731 rescued WM deficits and enhanced prefrontal cortex (PFC)–ventral hippocampus (vHPC) theta synchrony in male *Df(16)A*^*+/−*^ mice, but had no benefit in female mutants and impaired performance in wild-type (WT) females. Transcriptomic profiling of the postnatal PFC revealed previously unrecognized sex-by-genotype interactions in gene expression associated with the 22q11.2 deletion also implicating GSK3B-associated pathways. Notably, *Gsk3b* expression itself displayed opposing patterns in *Df(16)A*^*+/−*^ mice relative to WT mice, being elevated in males and reduced in females, potentially explaining the observed sex-specific behavioral and circuit responses. Our findings suggest that GSK3B is part of a broader, sexually dimorphic gene network that governs PFC circuit maturation and cognitive function. Specifically, our transcriptomic profiling delineates a postnatal window where the 22q11.2DS model exhibits these sexually dimorphic signatures. Notably, these signatures include many genes previously implicated in schizophrenia, autism, and intellectual disability, likely shaping the disorder’s sex-specific pathophysiology. More broadly, this work underscores the importance of incorporating sex as a biological variable in translational research and supports precision psychiatry approaches that align interventions with sex-specific neurobiological profiles.

## Introduction

Schizophrenia, like many neuropsychiatric disorders, exhibits significant sex differences in prevalence, age of onset, symptom profiles, and treatment response [1]. Understanding the biological basis of these differences is crucial for developing effective, and potentially sex-specific, therapeutic approaches. The 22q11.2 deletion syndrome (22q11.2DS) is a genetic disorder associated with ~20–30-fold increased risk of schizophrenia [2, 3]. Individuals with 22q11.2DS exhibit progressive impairments in prefrontal cortex (PFC)-dependent learning and cognitive functions, particularly working memory (WM) [4]. Despite the significant disease burden of 22q11.2DS, there are currently no approved pharmacological treatments. Mouse models of 22q11.2DS, such as the *Df(16)A*^*+/−*^ mouse, serve as valuable tools for investigating the underlying neurobiology and testing potential therapeutic strategies. The *Df(16)A*^*+/−*^ mouse model exhibits high construct validity due to the synteny between human chromosome 22 and mouse chromosome 16 [5–11].

The PFC is consistently implicated in the pathophysiology of schizophrenia [12, 13], with patients exhibiting deficits in PFC-dependent tasks assessing attention, WM, inhibitory control, and cognitive flexibility [14]. WM, a measure of PFC function across species [15], is significantly impaired in both individuals with 22q11.2DS and corresponding mouse models [7, 16]. Effective WM relies on the PFC’s ability to integrate and regulate information from various cortical and limbic structures [17, 18]. Disruptions in this integrative communication, particularly between the PFC and ventral hippocampus (vHPC), can lead to WM deficits [7, 19, 20]. In 22q11.2DS, altered long-range connectivity within this pathway [21, 22], coupled with dysregulation of synaptic plasticity in both the PFC and vHPC [23–26], likely contributes to these cognitive deficits. Restoring PFC-vHPC communication has been linked to improvements in WM in 22q11.2DS mouse models [27]. Similarly, adolescence represents a critical period for refinement of functional connectivity and optimization of information integration through synaptic plasticity in the PFC [22, 28, 29]. Genetic, pharmacological, or environmental disruptions during this period can lead to prefrontal excitatory-inhibitory imbalance, overall prefrontal hyperexcitability, and increased risk of schizophrenia later in life [30].

Converging evidence implicates altered glycogen synthase kinase 3 (GSK3) signaling as a common factor in the etiology of schizophrenia and other major psychiatric disorders [31–33], owing to its central role in the regulation of synaptic plasticity and neurodevelopment [22, 31, 34–37]. Supporting this, some antipsychotic medications, such as haloperidol and raclopride, indirectly inhibit GSK3 [38, 39], further motivating efforts to develop GSK3-targeted therapeutics. GSK3 exists as two distinct paralogs, GSK3A (GSK3-α 51 kDa) and GSK3B (GSK3-β 47 kDa), encoded by separate genes. While our previous work demonstrated that non-selective GSK3 inhibition could rescue WM deficits in male *Df(16)A*^*+/−*^mice [27] the present study focuses on GSK3B-paralog selective inhibition given its significantly improved safety profile [40]. Concomitant inhibition of both GSK3A and GSK3B can lead to increased nuclear translocation of β-catenin, raising safety concerns, especially for long-term treatments [41, 42], and highlighting the need to explore the therapeutic value of selective GSK3B modulation.

Investigating the effects of developmental, paralog-selective GSK3B inhibition, we identified a surprisingly robust sex-dependent divergence in the *Df(16)A*^*+/−*^ model. While developmental GSK3B inhibition rescued working memory deficits and restored PFC-vHPC communication in adult males, it provided no benefit to females and even impaired performance in WT female controls. Transcriptomic profiling around the developmental treatment window (P7–P27) revealed significant sex-by-genotype interactions, notably including opposing baseline *Gsk3b* expression patterns: upregulation in male mutants and downregulation in females. This sexually dimorphic landscape extended to core pathways involved in dopaminergic signaling and axon guidance, as well as high-confidence risk genes for schizophrenia and autism. Collectively, these results demonstrate that sex profoundly modulates prefrontal vulnerability and therapeutic response in this model, underscoring the necessity of integrating sex as a biological variable in neurodevelopmental research to advance precision medicine strategies.

## Results

### GSK3B but not GSK3A inhibition differentially affects working memory in male and female *Df(16)A*^*+/−*^ mice

Juvenile male and female *Df(16)A*^*+/−*^ and WT mice were treated with 10 injections (3 mg/kg) of either paralog-selective GSK3B (BRD3731) or GSK3A (BRD0705) inhibitors, or vehicle from postnatal day 7 (P7) to postnatal day 27 (P27). The treatment dose of 3 mg/kg was selected to balance therapeutic efficacy with long-term safety. This conservative strategy was adopted to minimize potential developmental toxicity during the chronic juvenile administration window (P7–P27). Furthermore, this dosage ensures direct comparability with the effective concentration of the previously used non-selective GSK3 inhibitor, SB-216763 (2 mg/kg) in this model [27]. Confirming significant brain penetration of the drugs, we measured mean BRD3731 and BRD0705 concentrations of 1337 and 1333 ng/g, respectively, in P7 brains (*n* = 12; Treatment as a factor for BRD3731: *p* < 0.0001, BRD0705: *p* = 0.0018, Supplementary Fig. 1A).

**Fig. 1:**
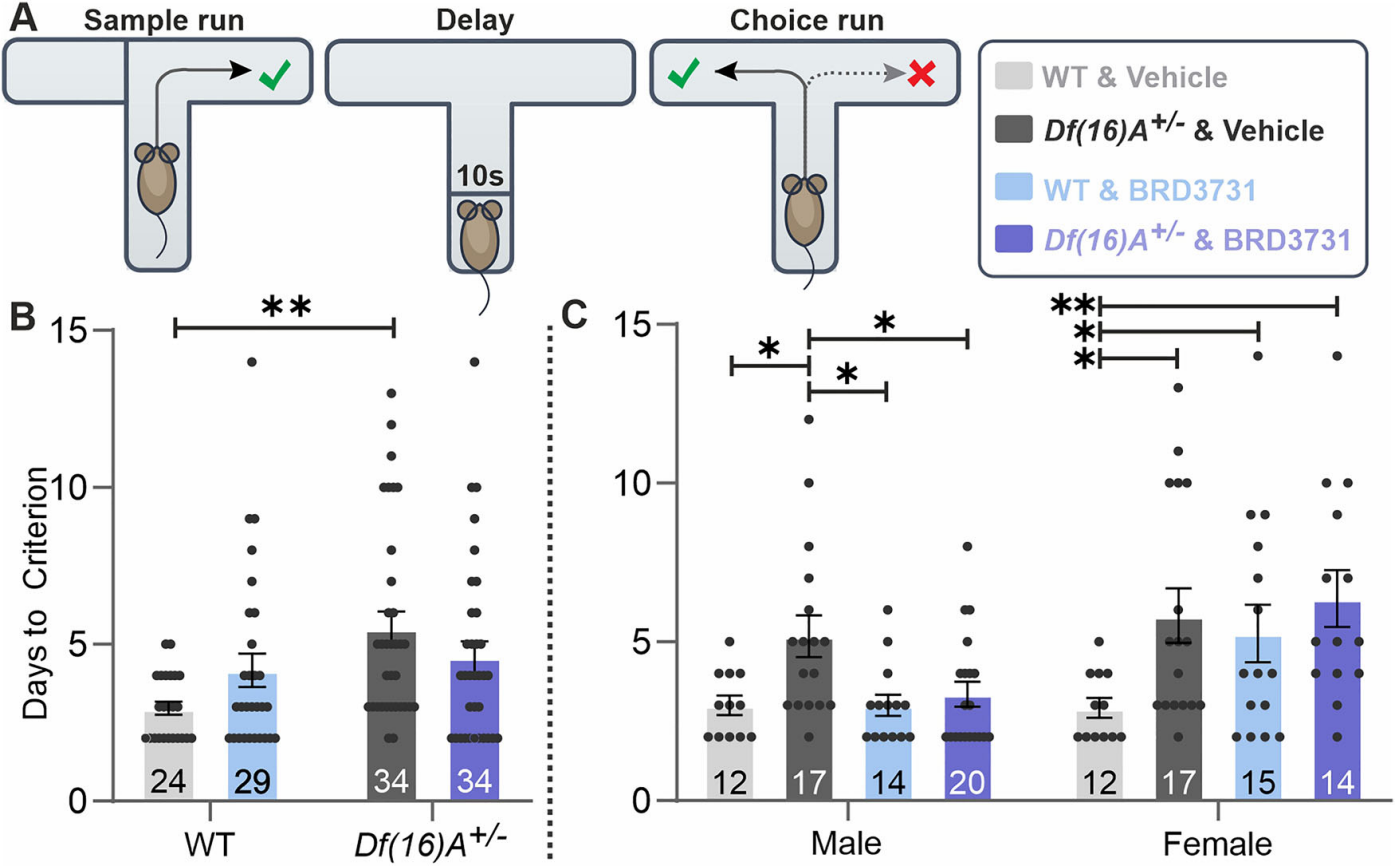
Sex-specific rescue of working memory deficits by developmental GSK3B inhibition in Df(16)A^+/−^ mice. **A** Schematic of the spatial working memory task. Mice were required to remember the previously visited arm after a 10-s delay and select the opposite arm to receive a reward. **B** Performance on the spatial working memory task, quantified as days to reach criterion. Initial two-way ANOVA (Genotype x Treatment, collapsed across sex) revealed a significant main effect of Genotype (*p* = 0.003), indicating impaired performance in *Df(16)A*^*+/−*^ mice, and a significant Genotype x Treatment interaction (*p* = 0.03). **C** Subsequent three-way ANOVA (Sex x Genotype x Treatment) revealed a significant main effect of Sex (*p* = 0.002) and, critically, a significant Sex x Treatment interaction (*p* = 0.01), indicating a sex-specific effect of BRD3731 (GSK3B inhibitor). While both male and female *Df(16)A*^*+/−*^ mice required more days to reach criterion than their respective WT controls (*p* = 0.04 and *p* = 0.01, respectively), only BRD3731-treated male *Df(16)A*^*+/−*^ mice showed improved performance (*p* = 0.04). BRD3731 treatment did not improve performance in female *Df(16)A*^*+/−*^ mice (*p* = 0.5) and impaired performance in WT female mice compared to vehicle-treated controls (*p* = 0.03). *p < 0.05, **p < 0.01, ***p < 0.001; mice per group indicated within each bar.

Mice were then trained in adulthood (3 months of age) in a spatial delayed non-match-to-sample WM task. The task required animals to remember a previously visited goal arm of a T-shaped maze during the first (“sample run”) half of each trial, and select the opposite arm to receive a reward during the second (“choice run”) half (Fig. 1A). Mice underwent daily training sessions of 10 trials per day until they performed the task correctly on 70% of the trials each day for three consecutive days, as in previous work [7, 27].

Initial analyses including both sexes confirmed the expected genotype-dependent WM task acquisition deficit, with *Df(16)A*^*+/−*^ mice taking longer to learn the task than WT controls (*p* = 0.003, Fig. 1B). A significant Genotype × Treatment interaction was observed for GSK3B inhibition with BRD3731 (*p* = 0.03), but post hoc comparisons did not yield statistically significant pairwise differences between drug- and vehicle-treated mice. However, incorporating sex as a factor revealed a significant sex-specific effect of BRD3731 (Sex x Treatment interaction: *p* = 0.01; Fig. 1C). Both male and female *Df(16)A*^*+/−*^ mice required more training days to reach criterion performance compared to WT controls (*p* = 0.04 and *p* = 0.01, respectively). Crucially, only male *Df(16)A*^*+/−*^ mice showed improved performance (*p* = 0.04) following treatment with BRD3731. In contrast, BRD3731 treatment did not rescue performance in female *Df(16)A*^*+/−*^ mice (*p* = 0.5). Moreover, BRD3731 treatment significantly impaired performance in WT female mice compared to vehicle-treated WT controls (*p* = 0.03). To confirm the effectiveness of our pharmacological intervention and ensure that the observed sex differences were not due to differential drug exposure, we analysed the brain concentration data considering Sex as a Factor in *Df(16)A*^*+/−*^ mice. We identified no significant differences in brain concentrations between male and female mice for BRD3731 (Sex as a factor: *p* = 0.26; Male vs. Female at 3 mg/kg: *p* = 0.13, Interaction Sex x Treatment: *p* = 0.28 Supplementary Fig. 1A).

To assess whether selective inhibition of GSK3A showed comparable effects, we tested a cohort of WT and *Df(16)A*^*+/−*^ mice on the WM task following developmental BRD0705 or Vehicle treatment. While we identified a significant main effect of Genotype (*p* = 0.0002), BRD0705 treatment had no significant effect, regardless of genotype or sex (Sex x Treatment *p* = 0.54, Sex x Genotype *p* = 0.65, Sex x Treatment x Genotype *p* = 0.07; 3-Way ANOVA, Supplementary Fig. 1B). There were no significant sex differences in the brain concentrations of BRD0705, similar to BRD3731 (Treatment as a factor *p* = 0.0018; Sex: *p* = 0.64. Interaction: *p* = 0.51; 2-way ANOVA, *n* = 12). To rule out general hyperactivity as a confounding factor for either experiment, we measured latency to reach the goal location; no significant differences were found between treatment and vehicle groups for either GSK3B or GSK3A inhibition (Supplementary Fig. 2).

### GSK3B inhibition differentially modulates PFC-vHPC theta coherence in male and female *Df(16)A*^*+/−*^ mice

**Fig. 2:**
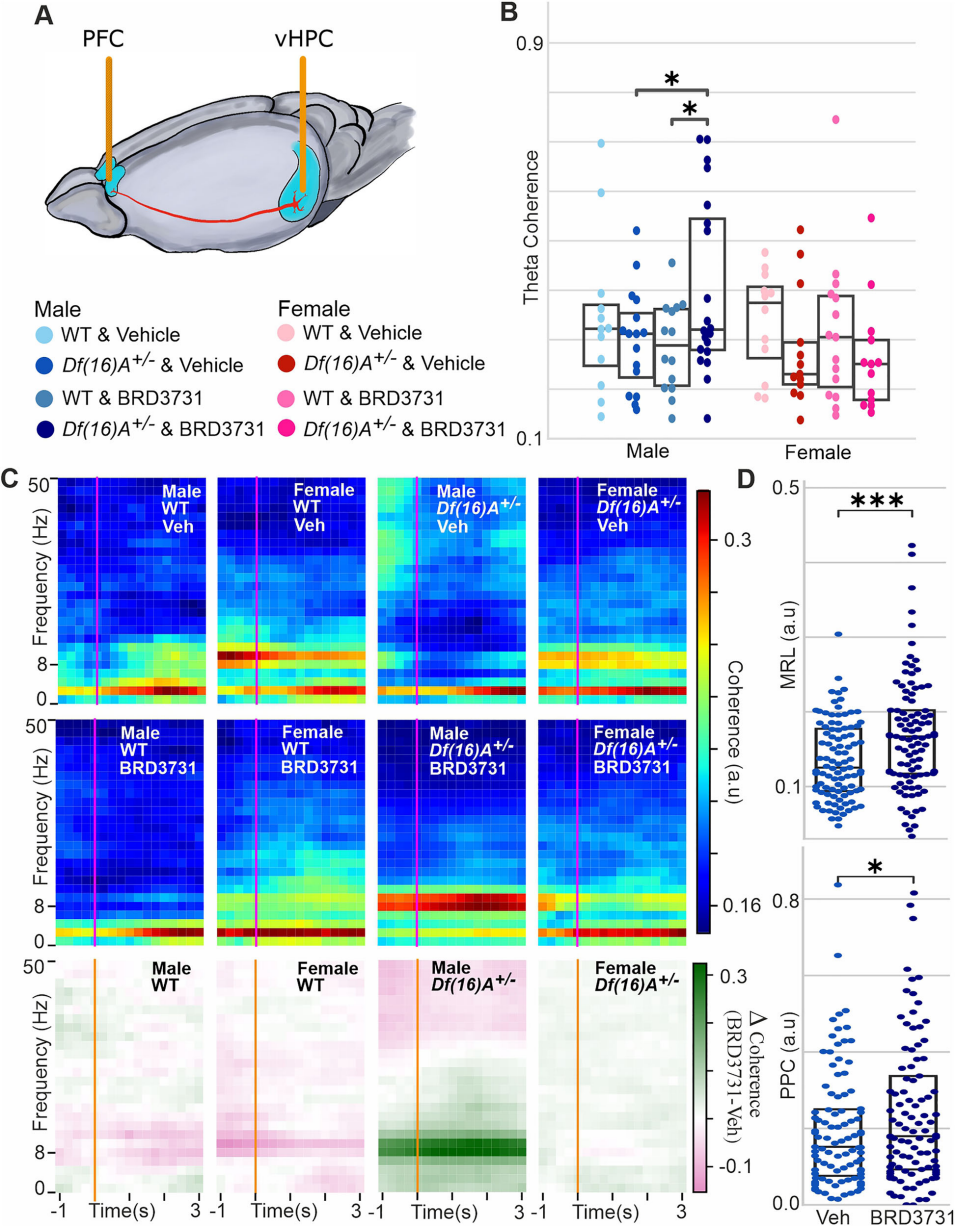
Developmental GSK3B inhibition treatment selectively enhances vHPC-PFC theta synchrony and neuronal phase-locking in male Df(16)A^+/−^ mice. **A** Schematic of electrode placement in the ventral hippocampus (vHPC) and PFC for local field potential (LFP) recordings. **B** vHPC-PFC theta coherence during goal runs. BRD3731 treatment significantly increased theta-band (7–12 Hz) coherence between the vHPC and PFC in male Df(16)A^+/−^ mice (*p* = 0.048) but not in female Df(16)A^+/−^ mice (*p* = 0.7). A three-way ANOVA (Genotype, Sex, Treatment, *n* = 115 animals) revealed a Genotype x Sex interaction (*p* = 0.016) as well as a Sex effect (*p* = 0.004), Sex x Treatment *p* = 0.09) (**C**) Time-frequency coherence plots from Df(16)A^+/−^ and WT mice without (top) or with BRD3731 (middle) treatment. Male vehicle (VEH)-treated Df(16)A^+/−^ mice show an absence of theta coherence, while male BRD3731-treated Df(16)A^+/−^ mice exhibit strong theta coherence around the onset of the goal run (vertical orange line indicates door opening). Bottom: Difference plot (BRD3731 - VEH) highlighting the treatment-induced increase (green) or decrease (magenta) in theta coherence in male compared to female Df(16)A^+/−^ and WT mice. Treated male Df(16)A^+/−^ show a strong increase in theta coherence. **D** BRD3731 treatment significantly increased the phase-locking of PFC single-unit activity to vHPC theta oscillations in male Df(16)A^+/−^ mice (*n* = 197 neurons: 95 VEH, 102 BRD3731; Pairwise Phase Consistency (PPC): *p* = 0.049; Mean Resultant Vector Length (MRL): *p* = 0.0002). *p < 0.05, ***p < 0.001.

One measure of inter-regional brain communication is the magnitude of phase synchronization (coherence) between local field potentials (LFPs) recorded in two or more brain areas. Because theta-band (7–12 Hz) coherence between the PFC and vHPC is a well-established cross-species correlate of WM task performance [43–45], we sought to corroborate our behavioral findings with neurophysiological data by examining PFC-vHPC coherence. We recorded LFPs from electrodes implanted in the vHPC and PFC (Fig. 2A) in the same animals used in the behavioral experiment to measure oscillatory synchrony between these structures.

We observed no significant overall reduction in theta coherence in either male or female *Df(16)A*^*+/−*^ mice compared to their WT littermates (*p* = 0.4 and *p* = 0.2, respectively). However, we found significant sex-specific effects of BRD3731 treatment on theta coherence (Fig. 2B, C; Supplementary Fig. 3). BRD3731 treatment increased theta coherence in male *Df(16)A*^*+/−*^ mice (*p* = 0.048) but not in female *Df(16)A*^*+/−*^ mice (*p* = 0.7) compared to their respective vehicle-treated controls (Fig. 2B, C). Furthermore, BRD3731-treated male *Df(16)A*^*+/−*^ mice exhibited significantly higher coherence than treated female *Df(16)A*^*+/−*^ mice (*p* = 0.017). We further analysed coherence in the beta range (20–30 Hz) but did not identify significant effects of sex, genotype or treatment, or any interactions (3-way ANOVA). Similarly, no significant main factor or interaction effects were identified for low-gamma coherence (30–50 Hz). Visualizations across a broad set of frequencies (2–50 Hz, Fig. 2C) indicated that this increase in coherence following BRD3731 treatment in male *Df(16)A*^*+/−*^ mice was specific to the theta range.

### GSK3B inhibition enhances phase-locking of PFC neurons to vHPC theta oscillations in male *Df(16)A*^*+/−*^ mice

We next investigated whether the observed increase in vHPC-PFC coherence in treated males extended to the single-neuron level. Based on the coherence findings, we hypothesized that BRD3731 treatment would increase the phase-locking of individual PFC neurons to vHPC theta oscillations (7–12 Hz). We recorded and identified PFC single units in both BRD3731-treated and vehicle-treated male *Df(16)A*^*+/−*^ mice. A total of 197 units (102 in treated and 95 in untreated mice) were analyzed. We quantified phase-locking using two complementary measures: mean resultant vector length (MRL) and pairwise phase consistency (PPC). Both measures confirmed that BRD3731 treatment significantly increased phase-locking in male *Df(16)A*^*+/−*^ mice (MRL: *p* = 0.0002; PPC: *p* = 0.049; *n* = 198; Fig. 2D). Taken together, these electrophysiological findings corroborate the behavioral results, and suggest that BRD3731-induced enhancement of vHPC-PFC neural synchrony in male *Df(16)A*^*+/−*^ mice may underlie their improved WM task performance.

### Transcriptomic analysis reveals novel sex- and genotype-dependent alterations in the developing PFC of *Df(16)A*^*+/−*^ mice

These behavioral and pharmacological results suggest that an underlying sexual dimorphism in the postnatal brain affects responsivity to GSK3B inhibition. To investigate the molecular basis of this dimorphism, we performed gene expression profiling of the PFC. Thousands of genes are differentially expressed in male and female brains during early postnatal stages, particularly in cortical regions that undergo extensive maturation during this period [46]. We hypothesized that differential gene expression during the P7-P28 period (P7 and P28 corresponding to the respective start and end of the GSK3B treatment regime) might provide a mechanistic explanation for the observed sex differences in treatment response. To investigate this, we performed a transcriptomic analysis of the PFC of untreated WT and *Df(16)A*^*+/−*^ mice at 1 week and 4 weeks of age.

Following quality control (see Methods, Supplementary Fig. 4A,D), we first confirmed the expected tripartite gene expression signature characteristic of *Df(16)A*^*+/−*^ mice. As anticipated, genes located within the 22q11.2 deletion region were downregulated by approximately 50% in *Df(16)A*^*+/−*^ mice compared to WT littermates at both P7 and P28 (Supplementary Fig. 4B,E). This dosage-dependent reduction was consistently observed across both sexes and developmental time points (Supplementary Fig. 5). In addition, we observed robust upregulation of *Emc10* and several non-coding RNAs, including primary transcripts of microRNAs and long non-coding RNAs such as *Mir9-2* and *Mir22hg*, in both male and female mice, in agreement with previous findings [47]; (Supplementary Fig. 5). As we were specifically interested in the interplay between sex and genotype, we performed a linear model-based differential gene expression analysis in PFC samples with two main factors (Sex and Genotype) and their interaction term (Genotype x Sex). In the analyses that follow, we focus specifically on genes that show significant Genotype x Sex differential expression within a given developmental time point. Differential expression results based on genotype or sex alone are provided in Supplementary Data 1 and Supplementary Fig. 5.

**Fig. 3:**
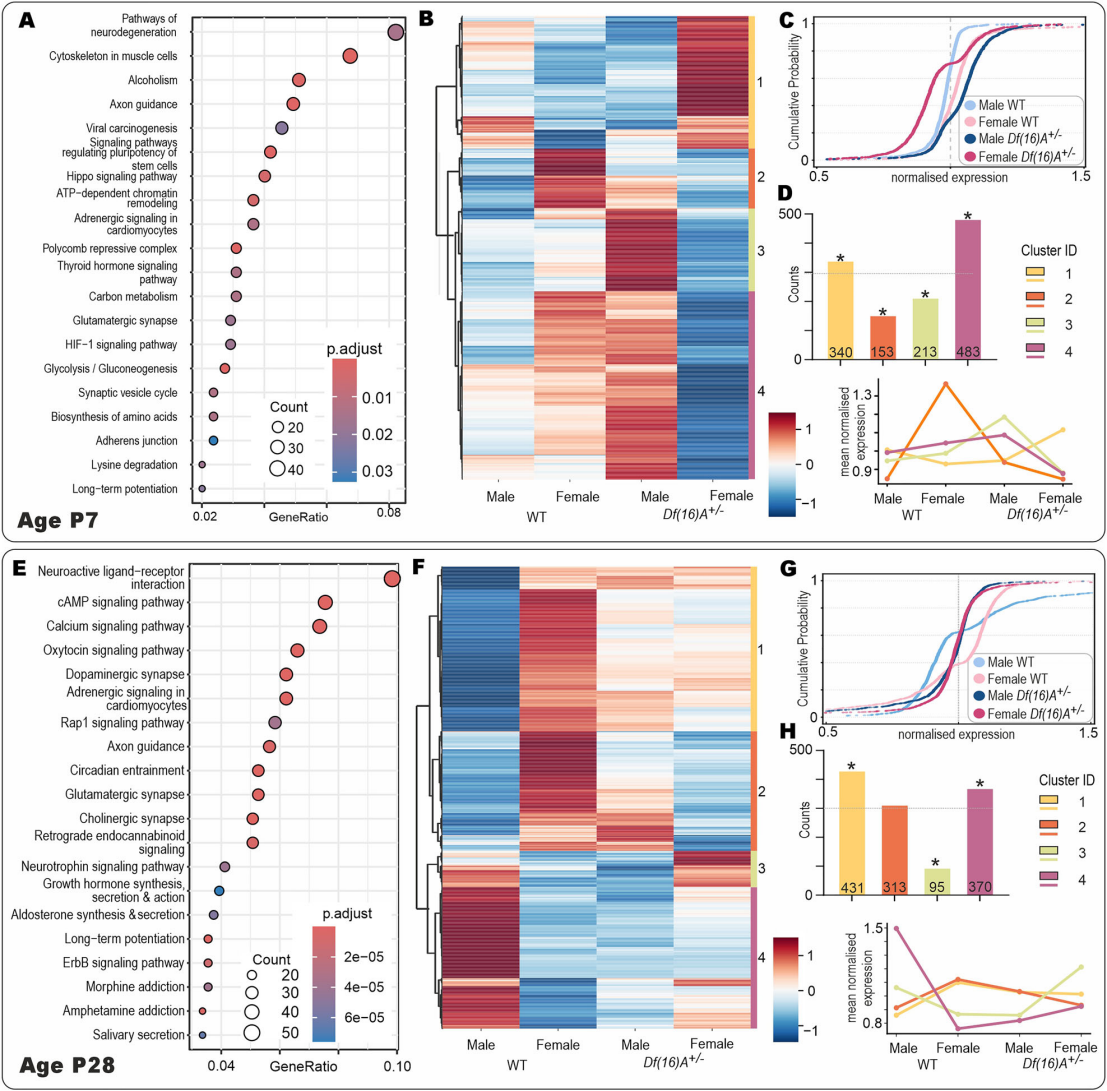
Genotype-Sex interaction gene expression profile in the PFC of 1- and 4-week-old WT and Df(16)A^+/−^ mice. **A** Over-representation analysis of the 1189 genes and in (**E**) of 1209 genes significantly modulated by the Genotype x Sex interaction at 1 and 4 weeks respectively, reveals enrichment particularly for signaling and synaptic pathways. Only the top 20 pathways are represented. Circle size represents the number of significant genes while color represents the level of significance. **B** and **F** Heatmap of normalised expression for all significant Genotype x Sex interaction DEGs across the four experimental groups, with hierarchical clustering representing the four main expression patterns (clusters 1–4). **C** and **G** Cumulative distribution plots of normalised expression values for genes within each of the four clusters across the experimental groups. At P7 all 4 groups are significantly different in their distribution (p.adj. < 0.0001, KW test). At P28 the variance is generally smaller with only the distributions between male and female WT mice, female WT and female *Df(16)A*^*+/−*^, and female WT and male *Df(16)A*^*+/−*^ being significantly different (KW test, p.adj < 0.0001). **D** The majority of genes and main clusters 1, 3 and 4 present pronounced sex-biased patterns particularly in *Df(16)A*^*+/−*^mice not observed in WT mice. In contrast, only the smallest cluster 2 has a pronounced increased expression in female WT mice compared to male WT mice, not observed in *Df(16)A*^*+/−*^ mice. **H** At 4 weeks most genes present increased expression in female WT compared to male WT (cluster 1 and 2); these differences are either absent (cluster 1) or reversed (cluster 2) for both sexes in the *Df(16)A*^*+/−*^ *group*. A minority of genes *(*95) is highly expressed in female *Df(16)A*^*+/−*^ compared to the other 4 experimental groups. Cluster 4 represents genes that are highly increased in male WT mice compared to female WT, but whose expression is comparable between female WT levels and male *Df(16)A*^*+/−*^ mice. Total χ² = 216.11 (P7) and χ² = 212.52 (P28) for D and H respectively; *p < 0.001.

We first identified 1189 differentially expressed genes (DEGs) showing significant Genotype × Sex interaction effects on expression at P7 (p.adj < 0.05; Fig. 3A–D, Supplementary Fig. 4C, Supplementary Data 1). Pathway enrichment analysis revealed that these DEGs are significantly overrepresented in key neurodevelopmental pathways, including axon guidance, Hippo signaling, adrenergic signaling, and pathways regulating the pluripotency of stem cells, among others (Fig. 3A). Strikingly, sex-biased expression differences were more pronounced in *Df(16)A*^*+/−*^ mice compared to WT mice (Fig. 3B, C; KW test p.adj < 0.0001), with the majority showing increased expression in male *Df(16)A*^*+/−*^ mice relative to their female counterparts (Fig. 3B–D). To better characterize these patterns, we performed hierarchical clustering and identified four prominent gene expression clusters (Fig. 3D). One cluster (Cluster 2) included genes with elevated expression in female WT mice compared to male WT mice, a sex-biased pattern that was lost in *Df(16)A*^*+/−*^ mice. In contrast, Clusters 3 and 4 encompassed the majority of genes with increased expression in male versus female *Df(16)A*^*+/−*^ mice; this sex-biased pattern was less pronounced in WT mice. Notably, most genes in these clusters also showed elevated expression in male *Df(16)A*^*+/−*^ mice compared to male WT mice. Cluster 4 exhibited the highest number of enriched pathways, including axon guidance, hippo signaling, Wnt signaling, and pathways regulating pluripotency of stems cells (Supplementary Fig. 6A).

Next, at 4 weeks of age, we identified 1209 DEGs showing a significant Genotype x Sex interaction (p.adj < 0.05; Fig. 3, Supplementary Fig. 4F, Supplementary Data 1). Over-representation analysis revealed that these genes were enriched in key neurodevelopmental and signaling pathways, including calcium, cAMP, oxytocin, dopaminergic, adrenergic, glutamatergic, endocannabinoid and cholinergic pathways (Fig. 3E). Visualization of these, 1209 genes revealed a disruption of WT sex-specific expression patterns in *Df(16)A*^*+/−*^ mice, indicating that the *Df(16)A*^*+/−*^ genotype alters normal sex-dependent transcriptional programs (Fig. 3F). As expected for genes identified through a Genotype × Sex interaction, nearly all pairwise group comparisons showed highly significant differences in expression (KS test, FDR correction, all p.adj. < 3.19 × 10^−33^; Fig. 3G). However, the direct comparison between male *Df(16)A*^*+/−*^ and female *Df(16)A*^*+/−*^ groups (KS test, p.adj = 0.0069) indicated that while sex differences persisted in *Df(16)A*^*+/−*^ mice, they were attenuated compared to the pronounced sex-specific expression patterns observed in WT animals at this developmental stage (Fig. 3F, G). Hierarchical cluster analysis (Fig. 3H; Chi-square test, χ² = 212.52, p~10^−45^) revealed that Cluster 1 and 2 represent genes with increased female-to-male expression in WT animals, and this pattern was either similar (Cluster 1) or reversed (Cluster 2) across sexes in *Df(16)A*^*+/−*^ animals. Cluster 1 and 2 encompassed genes with increased expression in male *Df(16)A*^*+/−*^ compared to male WT mice. Overrepresentation analysis of Cluster 1 genes included prominent pathways such as the dopaminergic synapse, pathways of neurodegeneration, MAPK signaling, ErB signaling, mTOR signaling, insulin resistance, and others (see Supplementary Fig. 6B). Cluster 3 represents 93 genes that, in contrast to WT mice, present higher expression in female *Df(16)A*^*+/−*^ compared to male *Df(16)A*^*+/−*^ mice. Cluster 4 comprises 370 genes that show markedly increased expression in male compared to female WT mice, and almost exclusively decreased expression in male *Df(16)A*^*+/−*^ mice. A significant number of risk genes for major neurodevelopmental disorders were identified among the Genotype × Sex interaction DEGs at P28, all sharing a common expression pattern. Specifically, we found 17 known syndromic autism spectrum disorder risk genes from the SFARI database, which were all assigned to clusters 1 and 2 (*p* = 0.0002; [49]; Supplementary Fig. 7). Likewise, three high-confidence schizophrenia risk genes (*Grin2a*, *Ash1l*, and *Sobp*) from the SCHEMA dataset (34 genes with q < 0.1 from [48] also showed this pattern, belonging exclusively to cluster 1 (*p* = 0.045; Supplementary Fig. 7). In all cases, the shared pattern was characterized by higher expression in WT females compared to WT males, a sex difference that was attenuated in *Df(16)A*^+/−^ animals. Overall, these findings demonstrate that by 4 weeks of age, the *Df(16)A*^*+/−*^ genotype profoundly reshapes sex-specific gene expression programs in the PFC, often attenuating or even reversing expression patterns typically observed in WT animals.

**Fig. 4:**
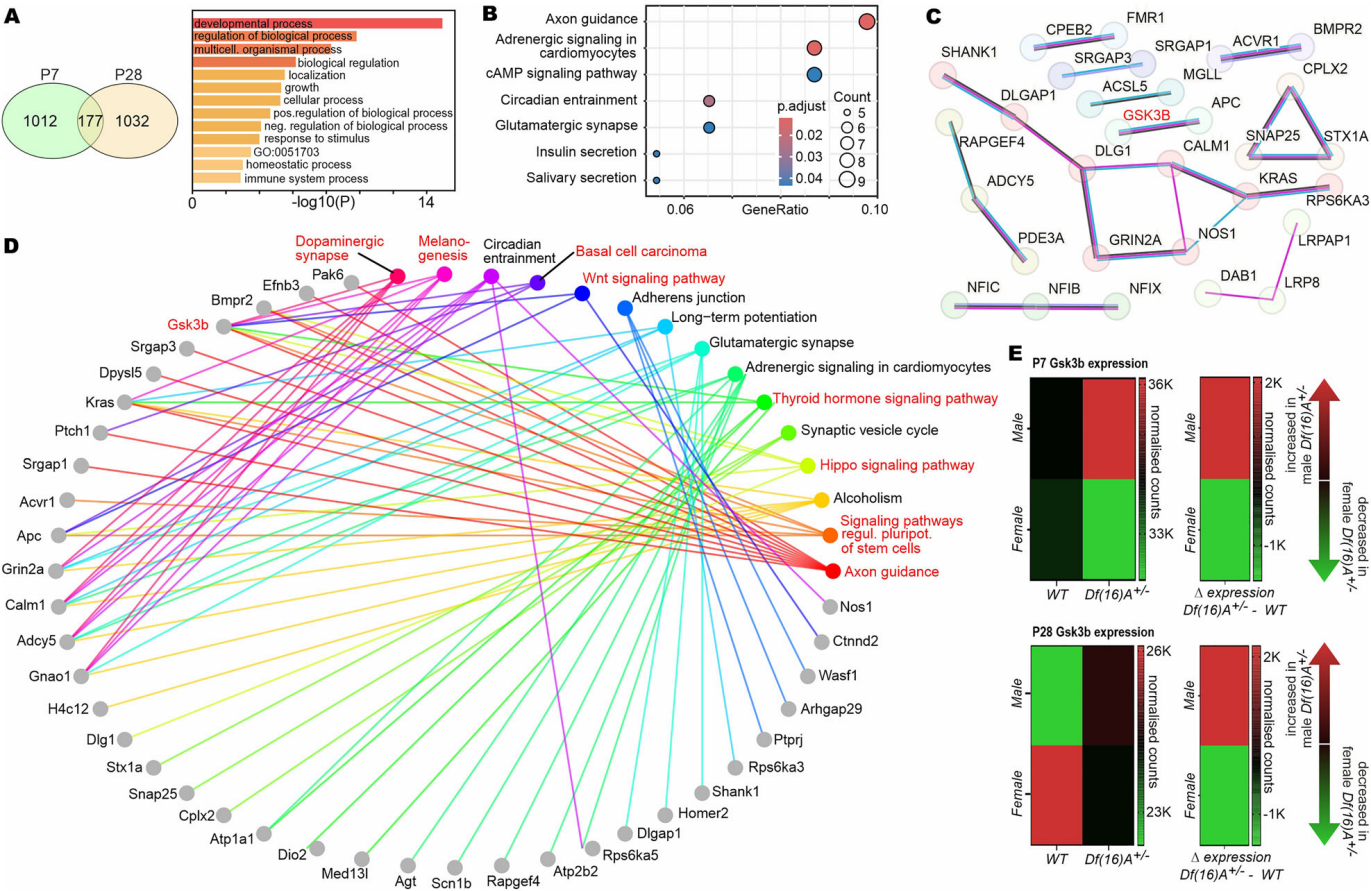
Significant overlapping pathways and genes based on Genotype x Sex Interaction at P7 and P28, with GSK3B as a critical node. In total, 177 genes are significant at both timepoints P7 and P28 (**A**) that represent major GO pathways related to development biological process. **B** Specifically, the genes are significantly overrepresented in pathways related to axon guidance, adrenergic and cAMP signaling, as well as glutamatergic synapse and others. **C** The largest PPI network constructed from these genes include autism spectrum disorder-associated risk genes *Shank1, Dlgap1, Dlg1*, and *Grin2a*. Beyond this network, several other significant Schizophrenia or autism-associated risk genes (*Adcy5, Fmr1, Nfix, Nfib, Srgap3, Rapgef4*, and *Rps6ka3)* are identified as part of other networks. **D** Enriched Pathways that are significant at both P7 and P28 including their associated significant genes. *Gsk3b* (in red) is associated with several critical enriched pathways at both developmental time points. **E** At both time points, *Gsk3b* expression is increased in male *Df(16)A*^*+/−*^ mice compared to WT males, while it is decreased in female *Df(16)A*^*+/−*^ mice compared to WT females.

To further investigate commonalities across early postnatal development, we examined DEGs showing significant Genotype × Sex interaction at both P7 and P28. This analysis identified 177 genes (Supplementary Data 3) with consistent interaction effects across both time points (Fig. 4A). This shared gene set includes 8 X-chromosome genes (*Apln*, *Fmr1*, *Iqsec2*, *Fgf13*, *Mbtps2*, *Tmem255a, Rps6ka3, Slitrk4)*, 168 autosomal genes *(*e.g., *Shank1*, *Tcf4*, *Stx1a*, *Kalrn*, *Dio2*, *Pou3f2*, *Zfp365*, *Snap25*), and one mitochondrial RNA gene (*Mt-rnr1*). Many of these genes are associated with sex-biased neurodevelopmental trajectories and implicated in neuropsychiatric disorders that display sex differences in prevalence or clinical presentation [48–53]. Enriched KEGG (Kyoto Encyclopedia of Genes and Genomes) pathways related to the 177 genes include axon guidance, adrenergic and cAMP signaling, and glutamatergic synapse pathways (Fig. 4B). The largest protein-protein interaction (PPI) network across the 177 genes highlighted a connected module of autism spectrum disorder-associated risk genes including *Shank1*, *Dlgap1*, *Dlg1*, and *Grin2a* (Fig. 4C). Beyond the PPI-identified genes, additional syndromic autism spectrum disorder risk genes [49] include *Arid1b, Atp1a1, Fbxo11, Fgf13, Fmr1, Foxg1, Iqsec2, Kmt2e, Med13l, Naa15, Nfib, Nfix, Pabpc1, Pou3f3, Rps6ka3, Tcf4*, and *Wasf1*. Also among the 177 genes were several schizophrenia risk genes, as identified through SCHEMA [48], including *Grin2a, Ash1l, Sobp, Nfic, Adcy5, Sidt1, Eif4g2, Mex3b, Bmpr2, Agap2, Ildr2, Pabpc1, Slitrk4, Ppargc1a, Dscaml1, Dlgap1*, and *Atp2b2*. These findings suggest that the 22q11.2 deletion interacts with sex to modulate the expression of a convergent set of risk genes relevant to 22q11.2DS and its comorbid neurodevelopmental disorders.

### Sex-bias in gene expression for GSK3B-mediated pathways in *Df(16)A*^*+/−*^ mice

In parallel, we also analyzed pathway-level convergence based on the respective significant DEGs at each time point. Of the 15 shared pathways enriched among DEGs at both P7 and P28, over half were associated with *Gsk3b*-related signaling (Fig. 4D). *Gsk3b* itself (but not *Gsk3a*) emerged as one of the 177 genes exhibiting significant Genotype x Sex interaction, with opposite expression changes in male and female *Df(16)A*^*+/−*^ mice (Fig. 4E; P7: *Gsk3b* p.adj = 0.006; *Gsk3a* p.adj = 0.44; P28: *Gsk3b* p.adj = 0.006, *Gsk3a* p.adj = 0.439). At both time points, *Gsk3b* expression was increased in male *Df(16)A*^*+/−*^ mice compared to male WT mice. In contrast, *Gsk3b* expression was decreased in female *Df(16)A*^*+/−*^ mice compared to their respective WT controls. The persistent, sex-divergent expression of *Gsk3b* across both time points, together with the sex-specific behavioral effects of GSK3B inhibition, prompted us to investigate *Gsk3b*-related molecular pathways more deeply as a potential mechanism underlying the observed sex-specific responses to *Gsk3b* inhibition.

**Fig. 5:**
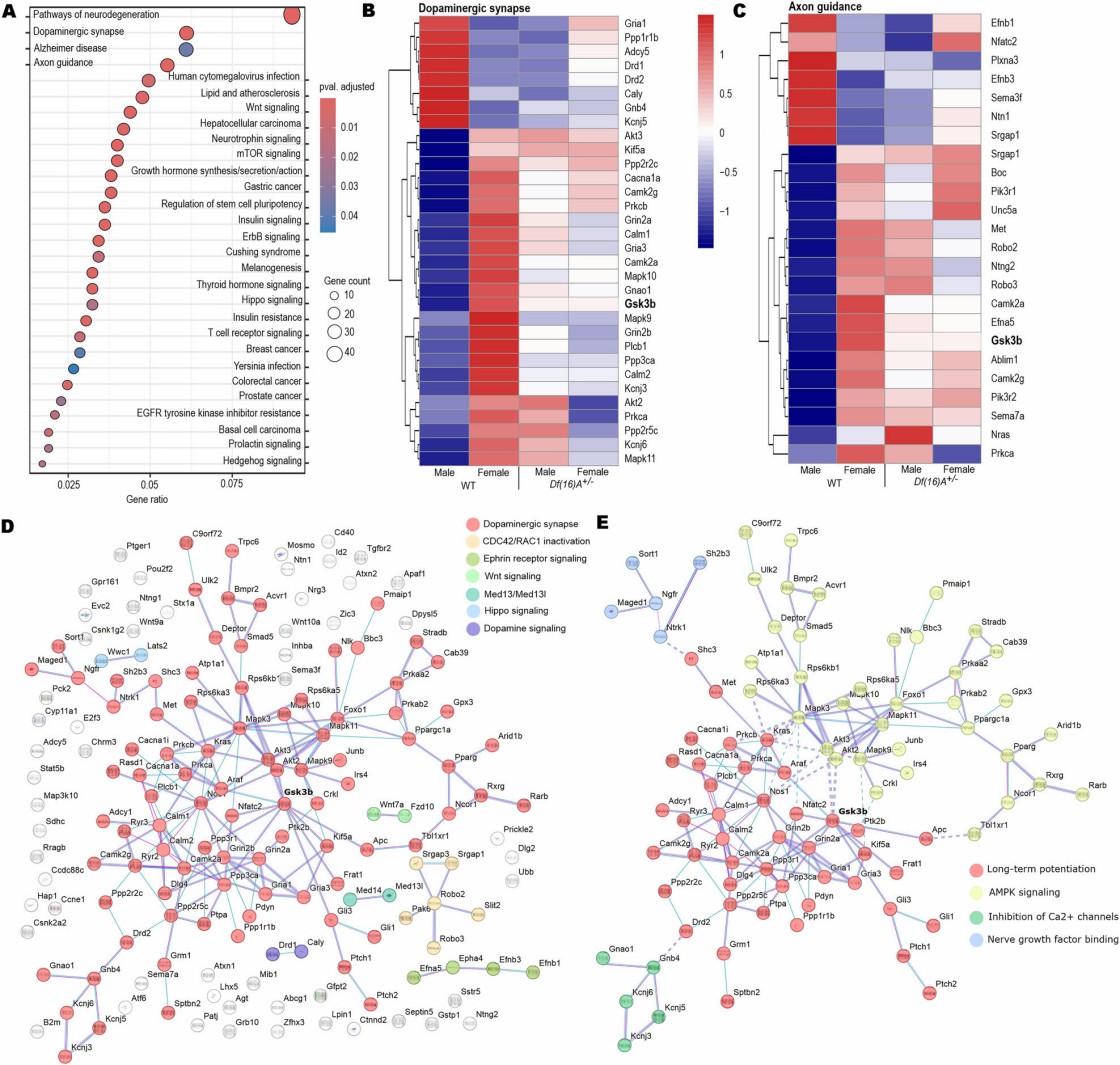
Sex- and genotype-dependent differential gene expression in the PFC of 4-week-old Df(16)A^+/−^ mice reveals significant over-representation of GSK3B in key pathways. **A** GSK3B-containing pathways identified by over-representation analysis. **B**, **C** Heatmaps of relative gene expression levels for DEG within the two most significant pathways: (**B**) dopaminergic synapse and (**C**) axon guidance. These heatmaps reveal a consistent pattern: *Gsk3b* expression is increased in male *Df(16)A*^*+/−*^ mice compared to WT males, while it is decreased in female *Df(16)A*^*+/−*^ mice compared to WT females. This pattern is also observed for a subset of other genes within these pathways. **D** Protein-protein interaction (PPI) network analysis (k-means clustering with k = 7) of all significant DEGs. GSK3B (in bold) occupies a prominent position within the largest identified network, which is centered on dopaminergic synapse function. **E** Sub-cluster analysis of the largest cluster in D (k-means clustering with k = 4). This analysis highlights additional pathways critical for synaptic and cognitive function. In (**D**) and (**E**), solid lines indicate both functional and physical protein associations: light blue lines represent known interactions from curated databases, purple lines represent experimentally determined interactions, and lavender lines represent interactions based on protein homology. Dashed lines follow the same color-coding and indicate connections between clusters. Colored circles of genes represent the respective pathways; white/transparent circles represent genes without a direct, significantly expressed interaction partner for one of the shown pathways.

We next focused our analysis specifically on pathways known to involve GSK3B. Using all significant DEGs, we aimed to uncover potential baseline molecular differences at each developmental time point that might underlie or modulate these divergent responses. At P28 the two most significant KEGG pathways were the dopaminergic synapse (p.adj = 1.8e–10) and axon guidance (p.adj = 2.9e–06) pathways (Fig. 5A), with notable DEGs like *Drd2* (p.adj = 0.009) and *Drd1* (p.adj = 0.001; Supplementary Data 1 and 2). Given the importance of dopaminergic synapse functioning (Fig. 5B) and axon guidance (Fig. 5C) in the pathophysiology of 22q11.2DS [24, 54], we further examined both pathways across all four experimental groups. For the dopaminergic synapse pathway, two main gene expression clusters were apparent (Fig. 5B). The first, smaller cluster showed decreased expression in WT females compared to WT males (8 genes). Most genes, including *Gsk3b*, showed increased baseline expression in WT females compared to WT males (24 genes). The *Df(16)A*^*+/−*^ genotype generally reversed these sex-specific expression profiles observed in WT mice for genes within these two clusters (Fig. 5B). Like for the dopaminergic pathway, genes within the axon guidance pathway (Fig. 5C) also displayed two main expression clusters, revealing baseline sex differences in WT mice that were modulated by genotype in a sex-dependent manner. Several other pathways associated with classical functions of GSK3B (e.g., mTOR signaling and Wnt signaling), as well as pathways related to growth hormone function, ERBB signaling, Hippo signaling, and insulin signaling, were also modulated (Fig. 5A; a complete overview of all significantly modulated pathways is reported in Supplementary Data 1).

To identify potential protein interaction networks associated with the observed gene expression changes in GSK3B (Fig. 5D, E), we performed a protein-protein interaction analysis (PPI) based on all significant DEGs for GSK3B-related pathways at P28. The dopaminergic synapse was identified as the largest interactive network, confirming our findings on the pathway enrichment analysis. This network included other significantly expressed direct interaction partners of GSK3B (APC, AKT2, AKT3, ARAF, GLI3, FRAT1, KIF5A, PPP3CA, PPP3R1, NFATC2). A sub-cluster analysis of this dopaminergic synapse-related network identified additional significantly modulated subnetworks related to AMPK and LTP signaling.

**Fig. 6:**
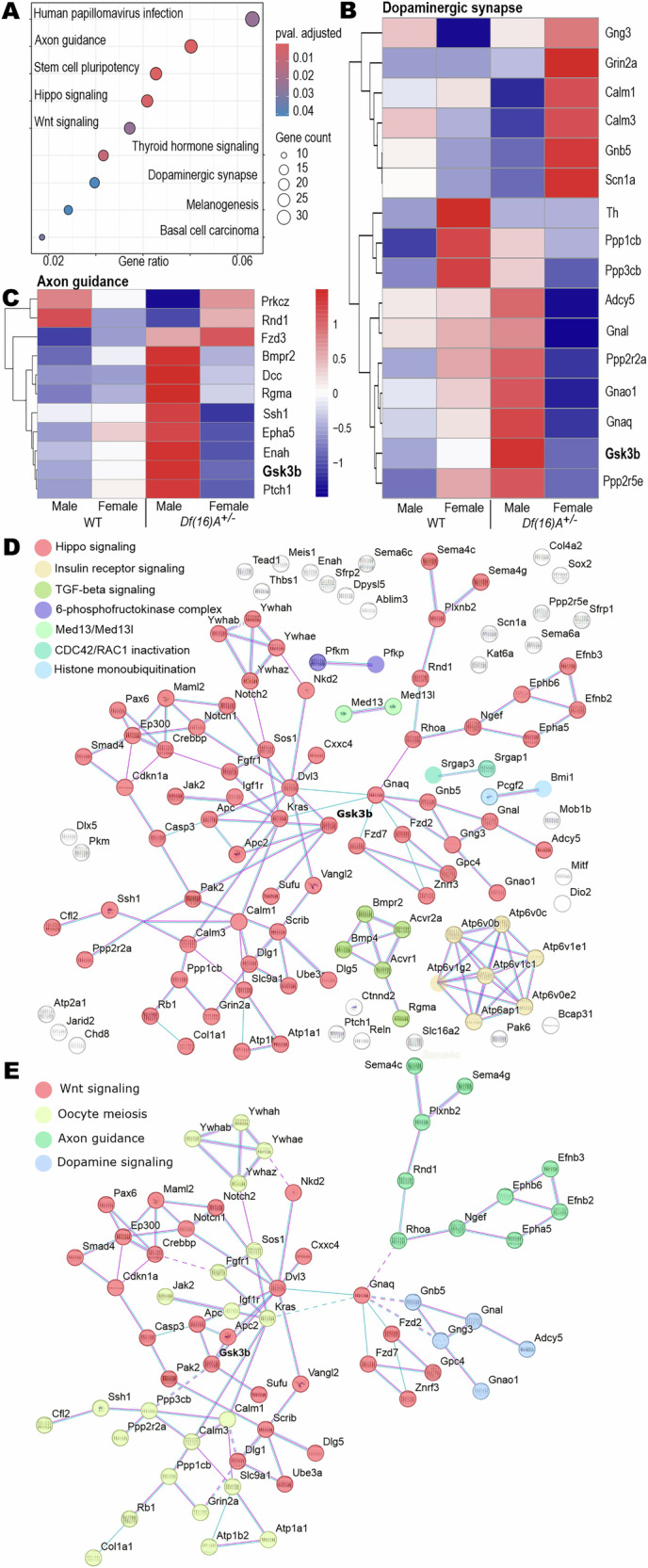
Sex- and genotype-dependent differential gene expression in the PFC of 1-week-old Df(16)A^+/−^ mice also shows significant over-representation of GSK3B, mirroring findings at 4 weeks. **A** GSK3B-containing pathways identified by over-representation analysis, presented as a dot plot of significantly enriched pathways. **B**, **C** Heatmaps of relative gene expression levels for DEGs within the (**B**) dopaminergic synapse and (**C**) axon guidance pathways. Like the 4-week-old mice, *Gsk3b* expression is increased in male *Df(16)A*^*+/−*^ mice compared to WT males, while it is decreased in female *Df(16)A*^*+/−*^ mice compared to WT females. Most significantly expressed genes within the axon guidance pathway (**C**) show increased expression in *Df(16)A*^*+/−*^ males compared to WT males and female littermates. **D** Protein-protein interaction (PPI) network analysis (k-means clustering with k = 7) of all significant DEGs, centered around GSK3B. **E** Sub-cluster analysis (k-means clustering with k = 4) of the largest PPI network (from panel D). In (**D**) and (**E**), solid lines indicate both functional and physical protein associations: light blue lines represent known interactions from curated databases, purple lines represent experimentally determined interactions, and lavender lines represent interactions based on protein homology. Dashed lines follow the same color-coding and indicate connections between clusters. Colored circles of genes represent the respective pathways; white/transparent circles represent genes without a direct, significantly expressed interaction partner for one of the shown pathways. Colored circles of genes represent the respective pathways; white/transparent circles represent genes without a direct, significantly expressed interaction partner for one of the shown pathways.

Although fewer *Gsk3b*-related pathways were significantly enriched based on the Genotype x Sex interaction at 1 compared to 4 weeks of age, both the axon guidance (p.adj = 2.2e–06) and dopaminergic synapse (p.adj = 0.04) pathways were again significant (Fig. 6A, Supplementary Data 2). In line with the overall differential gene expression findings for P7 (Fig. 3A), the sex differences in WT mice were less pronounced at P7 than in P28, while the differences between sexes in *Df(16)A*^*+/−*^ mice were more pronounced (Fig. 6B). As a result, the largest expression differences for the majority of significantly expressed genes within the dopaminergic and axon guidance these pathways (Fig. 6B, C) were observed between *Df(16)A*^*+/−*^ males and females, in line with earlier cluster-based findings (Fig. 3B, Supplementary Fig. 6A). Similarly, PPI network interaction analysis (Fig. 6D, E) identified pathways consistent with earlier findings across both age groups, including the Hippo signaling, axon guidance, dopaminergic, Wnt signaling, and other pathways. The expression of *Gsk3b* paralleled that of most differentially expressed genes in these pathways, with over-expression in *Df(16)A*^*+/−*^ males and under-expression in *Df(16)A*^*+/−*^ females compared to their sex-matched WT counterparts. This pattern, similar to that observed at P28, suggests opposing effects of the 22q11.2 deletion on *Gsk3b* expression in males and females, even at this early developmental stage. At P7, the Hippo pathway emerged as the largest interactive network centered around GSK3B, with APC, DVL3, KRAS, PAK2, and SUFU as direct interaction partners, which were also significantly modulated (Fig. 6D, E). The second-largest network, although not including GSK3B, was the insulin receptor signaling pathway. A sub-network analysis of the Hippo pathway-related network identified Wnt signaling, axon guidance, dopamine signaling, and oocyte meiosis as significant interaction networks.

## Discussion

This study investigated the potential of developmental, paralog-selective GSK3B inhibition as a therapeutic strategy for WM deficits in the *Df(16)A*^*+/−*^ mouse model of 22q11.2DS. Our findings reveal two unexpected and conceptually important observations. One is the pronounced sex difference in the behavioral and neurophysiological response to developmental GSK3B inhibition. While our previous work demonstrated that non-selective GSK3 inhibition rescues WM deficits in male *Df(16)A*^*+/–*^ mice [27], the present study shows that selective GSK3B inhibition is beneficial only in males and is, in contrast, detrimental to WM performance in WT females. Second, at the molecular level, transcriptomic profiling revealed that the 22q11.2 deletion disrupts normal sex-specific gene expression trajectories in the PFC, often attenuating or reversing WT patterns, consistent with early-onset and persistent sexually dimorphic transcriptional dysregulation. Across two timepoints in early postnatal development, we observed significant sex- and genotype-dependent alterations in multiple neuropsychiatric risk genes and GSK3B-regulated pathways. Together, these findings identify sex as a critical modifier of both molecular pathology and therapeutic response in 22q11.2DS, and refine how GSK3B-related mechanisms should be interpreted in neurodevelopmental disease.

### Sex-dependent GSK3B dosage governs circuit maturation and working memory

Notably, *Gsk3b* itself showed overexpression in male *Df(16)A*^*+/–*^ mice and underexpression in female *Df(16)A*^*+/–*^ mice, providing a plausible molecular substrate for the sex-specific behavioral and electrophysiological effects of paralog-selective GSK3B inhibition. In males, GSK3B inhibition may normalize abnormally elevated Gsk3b levels toward a physiological range, contributing to phenotypic rescue. In contrast, in WT females, pharmacological inhibition likely reduces GSK3B, effectively mimicking the already diminished baseline observed in female *Df(16)A*^*+/–*^ mice and resulting in impaired WM performance. In female *Df(16)A*^*+/–*^ mice, where *Gsk3b* expression is already reduced, further inhibition may be constrained by a floor effect, precluding therapeutic benefit. Together, these findings suggest that there is a critical range of GSK3B activity required for normal development of the circuits underlying working memory in line with earlier findings [55]. The deletion drives this activity in opposite directions in the two sexes, normalizing activity in males but driving it further from this range in females. These findings underscore the importance of considering sex as a biological variable in developing therapies for neurodevelopmental syndromes. Mechanistically, this sex-dependent dosage sensitivity likely reflects the central role of GSK3B in shaping circuit maturation. Given that GSK3B is a highly pleiotropic kinase, its developmental effects likely emerge through multiple interacting pathways, including developmental axon guidance and connectivity, dopaminergic signaling, synaptic plasticity, and oscillatory dynamics that together shape the observed behavioral and physiological outcomes.

GSK3B modulates axon growth and guidance, processes essential for establishing proper neuronal connectivity during development [56–61], whose disruption has been implicated in schizophrenia, 22q11.2DS, and WM deficits [24, 62]. Haploinsufficiency of *Zdhhc8*, a gene within the 22q11.2 locus, impairs palmitoylation of proteins regulating axonal growth and branching via Cdc42-dependent GSK3B signaling at the axonal tip [24]. Consistent with reports of reduced global and long-range connectivity reported in 22q11.2 deletion carriers [63–65], mouse models show decreased axonal projections and terminal arborization of vHPC pyramidal neurons to the PFC [24]. In line with this role of GSK3B in axon outgrowth and connectivity, behavioral improvements observed in male *Df(16)A*^*+/–*^ mice were accompanied by enhanced theta-band coherence between the PFC and vHPC, as well as increased phase-locking of PFC neurons to vHPC theta oscillations, without corresponding changes in beta or low-gamma bands. These effects were similar to those we observed previously with the non-specific GSK3 antagonist [27], suggesting similar neural mechanisms underly the similar behavioral effects in males. The PFC-vHPC circuit is critical for WM across species [45, 66], with theta oscillations coordinating neuronal activity and facilitating interregional communication [44]. The sex-specific effects, coupled with our observation of the opposite effects of the mutation on *Gsk3b* transcript levels, support the notion that appropriate levels of GSK3B are crucial for the proper development of the vHPC-PFC connectivity that supports WM.

GSK3B also interacts closely with dopaminergic signaling. D2R is a major upstream regulator of GSK3B activity [67–69], and overactive GSK3B in male *Df(16)A*^*+/–*^ mice, consistent with hyperdopaminergic PFC states, can inhibit NMDA receptor function, particularly by reducing GluN2B-mediated ionic currents [69]. In both 22q11.2 deletion carriers and *Df(16)A*^*+/–*^ mice, reduced COMT dosage has been implicated in hyperdopaminergic PFC states, although additional genes and downstream adaptations likely contribute to broader dopamine dysregulation [54, 70–72]. Developmental sex differences in dopamine signaling [73] may therefore confer differential sensitivity of male and female PFC circuits to both the 22q11.2 deletion and GSK3B inhibition. Consistent with this, at P28 we observed altered PFC expression of *Drd1* and *Drd2*, receptors critical for PFC function and WM [74–78], and whose dysregulation may promote excitation–inhibition imbalance in 22q11.2DS [79]. Together with opposing Gsk3b expression patterns, these findings support a sex-dependent interaction between dopamine signaling and GSK3B activity. In line with evidence that GSK3B inhibition can counteract dopamine hyperactivity [67, 80], inhibition may restore signaling balance in males but be ineffective (or detrimental) in females with already reduced Gsk3b expression.

Finally, GSK3B dysregulation impacts synaptic plasticity and oscillatory dynamics. GSK3B is a key regulator of neural oscillations underlying cognition, and its overactivity likely destabilizes oscillatory synchrony by altering AMPA and NMDA receptor and Ca_v_ and K_v_ channel function required for precise synaptic timing [81]. Transcriptomic analyses revealed changes in *Gria1, Gria3, Grin2a, Grin2b, Kcnj3, Kcnj5, Cacna1a, Camk2a*, and *Camk2g* (Fig. 5B–E; Fig. 6B, E), implicating pathways governing LTD, NMDA receptor signaling, Ca²⁺ dynamics, and AMPK-related processes. Increased GSK3B activation enhances NMDAR membrane expression and biases synapses toward LTD while reducing LTP or shifting its induction threshold, changes expected to impair network oscillations and contribute to cognitive dysfunction [36, 82–84].

Importantly, while multiple translational studies have implicated GSK3B in psychiatric disease [85–90], most have focused on adult pharmacological manipulation or global genetic models. Few studies have directly linked developmental GSK3B modulation to long-range circuit synchrony, and none, to our knowledge, have demonstrated a sex-dependent reversal of therapeutic direction at the level of behavior and interregional connectivity. Our findings therefore extend prior work by demonstrating that the timing, paralog specificity, and sex-specific molecular context of GSK3B modulation are decisive determinants of circuit and behavioral outcomes.

### Novel identification of sex-divergent risk genes during a critical period

The investigated period between P7-P28 in mice corresponds to a critical period of PFC development characterized by significant synaptic refinement and maturation of functional connectivity [28, 91]. Our findings highlight this period as a window of vulnerability to genetic and pharmacological perturbations, with potentially long-lasting consequences for cognitive function. The fact that we observed significant gene expression changes at both P7 and P28, with notable consistency in the affected pathways (Fig. 5), suggests that the underlying molecular mechanisms are established early in development and persist through this critical period. Among the 177 significant overlapping DEGs modulated by Sex and Genotype at both time points, a significant portion are linked to neurodevelopmental disorder phenotypes relevant for intellectual disability, autism spectrum disorder, attention deficit hyperactivity disorder, and schizophrenia, all highly clinically relevant for 22q11.2 deletion carriers. Within these 177 significantly expressed genes, we identified not only X-linked risk genes (including *Fmr1*, *Iqsec2*, *Fgf13*, *Tmem255a, Rps6ka3*), but also multiple autosomal genes such as *Arid1b, Atp1a1, Fbxo11, Foxg1, Kmt2e, Med13l, Nfib, Nfix, Pabpc1, Pou3f3, Wasf1, Shank1*, *Stx1a*, *Kalrn*, *Dio2*, *Pou3f2*, *Zfp365*, *Snap25*, and *Tcf4* [48, 49, 92–99]. This is the first time that many of these intellectual disability disorder, schizophrenia, and autism spectrum disorder risk genes are associated with the *Df(16)A*^*+/−*^ phenotype. For example, *Med13l* haploinsufficiency syndrome bears strong phenotypic resemblance to the 22q11.2DS, yet direct molecular links have not previously been established so far [100, 101]. Similarly, the ARID1B-associated Syndrome (also Coffin-Siris-Syndrome) shares overlapping phenotypic features, among them intellectual disability and autism spectrum symptoms [102]. Interestingly, all of the 17 significant syndromic autism spectrum disorder genes (*Arid1b, Atp1a1, Fbxo11, Fgf13, Fmr1, Foxg1, Iqsec2, Kmt2e, Med13l, Naa15, Nfib, Nfix, Pabpc1, Pou3f3, Rps6ka3, Tcf4, Wasf1*, Supplementary Fig. 7) exhibit higher female-to-male expression in WT mice, a pattern that is attenuated in *Df(16)A*^*+/−*^ mice. Similarly, for notable schizophrenia and/or intellectual disability disorder risk genes (*Grin2a, Ash1l, Sobp*), an almost identical pattern of sex-biased dysregulation emerges. As a result, earlier studies performed only in males, at the whole-brain level, or outside of this developmental window, may have failed to detect or implicate these risk genes in the etiology of 22q11.2DS.

### Limitations and future directions

While BRD3731 is a relatively selective GSK3B inhibitor, potential off-target effects cannot be completely ruled out. Despite equivalent brain drug exposure across sexes, potential sex-specific differences in binding affinity or downstream signaling kinetics may still exist. While these inhibitors showed no sex-dependent potency in human samples [40], the reduced baseline *Gsk3b* levels in the female PFC could create a distinct stoichiometric context that fundamentally alters the response to antagonism compared to the male PFC. Furthermore, bulk RNA-seq provides an average gene expression profile across many cell types. Future studies should focus on a protracted multiomic characterization, use single-cell RNA-seq to identify cell-type-specific changes in gene expression or employ cell-type-specific genetic manipulations of *Gsk3b* or downstream target pathways to causally test the proposed mechanisms as our current findings are primarily correlative in nature. Despite non-hormonal influences on the dopaminergic system early in life [103–105], our results warrant future research to investigate the role of sex hormones in mediating the observed sex-specific effects. Gonadectomy and hormone replacement experiments could help determine whether the sex differences are driven by organizational effects (long-lasting structural and functional changes) of hormones during development or activational effects that are more transient and emerge in adulthood.

In conclusion, this study demonstrates a sex-dependent impact of developmental GSK3B inhibition on WM and PFC-vHPC circuitry in a mouse model of 22q11.2DS. The divergent patterns of *Gsk3b* expression in male and female *Df(16)A*^*+/−*^ mice offers a direct molecular candidate for the divergent treatment outcomes. This core alteration, combined with the profound sex-specific dysregulation of critical neurodevelopmental pathways, provides a compelling framework for the sex-biased behavioral and circuit-level phenotypes we observe. Our findings underscore the importance of considering sex in translational research and advance the rationale for precision psychiatry approaches that stratify treatment strategies based on sex-specific molecular and circuit-level mechanisms.

## Materials and methods

### Animals and drug treatment

*Df(16)A*^*+/−*^ mice (RRID: MGI_3802827) and their wild-type (WT) littermates were generated from crosses of *Df(16)A*^*+/−*^ mice on a C57BL/6 J background [11] with C57BL/6 J mice (Jackson Laboratories, Bar Harbor, ME). Mice were group-housed (up to 5 mice per cage) in a temperature- and humidity-controlled facility under a 12-h light-dark cycle. Except during food restriction for behavioral testing, mice had ad libitum access to food and water. All animal procedures were approved by the National Institute of Neurological Disorders and Stroke and Columbia University Animal Care and Use Committee and were performed (ASP # 1403-17) in accordance with the National Research Council Guide for the Care and Use of Laboratory Animals. A total of 316 mice served as experimental subjects in this study.

BRD3731 and BRD0705 were obtained through a collaboration with Dr. Florence Wagner, Broad Institute, Havard University; [40]. The injection procedure followed previously established protocols [27]. Mice were injected intraperitoneally (i.p.) with either BRD3731 or BRD0705 (3 mg/kg) or vehicle (10% DMSO [ATCC® 4-X], 45% PEG-400 [VWR], 45% saline [VWR]) every other day (in the morning) from postnatal day 7 (P7) to P27, for a total of 10 injections, using a 28 G needle. The 3 mg/kg dose and P7-P27 treatment window were selected to balance therapeutic efficacy with safety during a sensitive developmental period, adopting a conservative strategy to mitigate the risk of cumulative toxicity in line with Passecker et al. 2025 and comparable to the 2 mg/kg dose of the non-selective inhibitor SB-216763 [27, 106]. The spatial WM task protocol commenced at 3 months of age. For the brain concentration measurements brains (P7) were collected 1 hr post i.p. injection and analyzed by Agilux Biolabs (USA) by a residual gas analyzer.

### Surgical procedure

3-month old mice were anesthetized with isoflurane (Butler Schein, Chicago, IL) and placed in a stereotaxic frame. A bundle of 6–11 twisted-wire stereotrodes (12.5 μm diameter; California Fine Wire Co., CA) were implanted in the medial PFC (1.65 mm anterior to bregma, 0.5 mm lateral to midline, 1.4 mm below brain surface; California Fine Wire Co., CA) and 2–3 single tungsten wire field electrodes (75 μm diameter) were implanted. One targeting the dHPC (1.94 mm posterior, 1.5 mm lateral, 1.3 mm ventral) and 1–2 targeting vHPC (3.16 mm posterior, 3.3 mm lateral, 4.5–5.0 mm ventral). 3–4 screws were attached to the skull and one screw over the cerebellum served as ground. All wires were connected to a multi-channel interface board (Blackrock) anchored to a 3D-printed microdrive. Mice were transcardially perfused with PBS, followed by 4% paraformaldehyde (PFA) in PBS. Brains were fixed in 4% PFA at 4 °C overnight and cryoprotected in 30% phosphate-buffered sucrose for 3 days at 4 °C. Brains were sectioned (40 μm) using a cryostat and mounted with DAPI Fluoromount-G mounting medium (Southern Biotech, Cat. #: 0100-20). Only recordings from verified electrode placements were included in the analyses.

### Spatial working memory task

Prior to behavioral testing on the T-maze spatial WM task, mice underwent food restriction for one week to achieve 85–90% of their body weights at the onset of restriction. During this period, mice were also habituated to handling by the experimenter. The automated T-maze consisted of a stem and two arms surrounded by 30 cm-high walls. During the first two days of behavioral training, mice were placed in the maze and allowed to freely explore for 30 min. Liquid reward (20% sweetened condensed milk diluted in deionized water) was delivered at the end of each arm (~50 μl in the start arm, ~100 μl in the goal arms) through a blunt 25 G needle. Mice received reward following alternate arm visits. Mice then underwent two consecutive daily sessions of shaping trials. During these trials, only one arm (left or right, randomized) was accessible, and mice received a milk reward for entering that arm. They then returned to the start box for another milk reward, and after a 10-s delay, the opposite arm was made accessible (forced choice). Shaping trials had a 20-s inter-trial interval, and sessions consisted of up to 30 trials over a 30-min period. On subsequent days, mice were trained on the spatial WM task. Each trial consisted of a “sample run” and a “choice run.” During the sample run, the mouse was allowed to explore one arm of the T-maze. After returning to the start area, a 10-s delay was imposed. During the choice run, the mouse had to choose the arm opposite to the one visited during the sample run to receive a reward. Following the choice run, the mouse returned to the start box and was confined there for 20 s before the next trial began. Pneumatic sliding doors controlled access to the start area and the arms. A circular rotary platform, with one half comprising the choice point of the maze, rotated 180° during the delay period of each trial to prevent mice from using scent cues to inform their choices. The T-maze was cleaned with a 70% ethanol solution between mice. Mice were trained for 10 trials per day until they reached a criterion of ≥ 70% correct choices for three consecutive days, or for a maximum of 14 training days. Animals in the BRD3731 experiment were always tethered to their implants when on the maze as we recorded electrophysiological signals during the task. Animals in the BRD0705 experiment were not implanted and not tethered.

### BULK-RNA sample collection & data processing

Following euthanasia and brain extraction at P7 and P28, PFC samples were collected on dry ice and snap-frozen in −50 degrees 2-methylbutane. Total RNA was extracted using Qiagen Plus Micro and Mini Kits, following standard protocols. RNA quality and quantity were assessed using an Agilent 2100 Bioanalyzer with RNA LabChip® kits (Agilent). Samples with RNA Integrity Numbers (RIN) above 9 and a total RNA amount of at least 500 ng were used for library preparation. Samples were sequenced on a single Illumina NovaSeq 6000 system S4 (8000 M) chip using 200 cycles, 100-bp paired-end reads, and a poly(A)-enriched library.

Raw sequencing data (fastq files) were quality-checked using FASTQC. Reads were aligned to the mm10 mouse reference genome using the STAR aligner. Four samples with less than 50% coverage or fewer than 20 million reads were excluded. The remaining samples had an average unique read percentage of 73% and a mean of 47 million reads. Gene-level counts were computed using featureCounts. Gene counts were transformed into transcripts per million (TPM) using RNAseqQC. BigWig files were generated for visual inspection, and principal component analysis (PCA) of the mapped reads did not reveal any biased clustering based on the main experimental variables (sex, genotype, or age).

RIKEN, MSTRG, and Gm genes were removed from further analysis to reduce the number of false negatives (or false positives). For subgroup analyses, genes with a row mean below 10 were filtered out. Following exploratory analysis and quality control, one additional sample was removed based on sample-to-sample distance calculations; the median of its correlation coefficients was an outlier (0.07 compared to a range of 0.21–0.58 for the other samples)

Differential expression analyses were performed using DESeq2 v1.47.1 [107], after removing unwanted variation using residuals from RUVSeq v1.36.0 [108]. The number of factors of unwanted variation (k-values) was set to nine and two for the P7 and P28 groups, respectively. Statistical significance testing was based on a Genotype:Sex interaction model. Genes with a Benjamini-Hochberg adjusted *p*-value below 0.05 were considered differentially expressed. Over-representation analysis was performed using clusterProfiler v4.10.0 [109] to explore the biological relevance of DEGs Significance for over-representation analysis was based on a false discovery rate (FDR)-adjusted *p*-value of 0.05. Protein-protein interaction (PPI) analysis was performed using the STRING database v12.0 [110] to visualize GSK3B network partners. K-means clustering of the interaction networks was performed using differentially expressed genes from GSK3B-containing, enriched pathways, mapped to human proteins. Only experimental data and databases were used as sources, and the minimum required interaction score was set to 0.7 (high confidence). Row-based scaling was used for heatmap plotting of gene counts within enriched pathways. Statistical tests of Fig. 3D and H were based on Kruskal-Wallis testing between the four cluster groups.

### Behavioral data analysis

Performance was measured as the number of correct trials divided by the total number of trials per session. Animal speed and distance were quantified using automated video tracking of body position and implant sensors during behavioral sessions. The sample sizes for these measures (Supplementary Fig. 2) vary slightly from the primary behavioral figures due to intermittent technical limitations in the tracking software for a small subset of trials, which precluded the accurate extraction of spatial coordinates. Behavioral events and trial information were obtained from event-triggered synchronization signals processed through a custom DataJoint data analysis pipeline. If not stated otherwise, 3-way ANOVA (sex, genotype, treatment as main factors; incl. all interaction effects) were performed to test significance. Normality testing was performed via the D’agostino & Pearson test [111] and quartiles visualised on a Q-Q plot. Corrections for multiple comparissons were controlled by FDR applying the 2-stage setup method of Benjamini, Krieger and Yekutieli [112]. Adjusted *p*-values are reported if not otherwise stated. Statistical Analysis was performed with Graphpad Prism v9. No animals were excluded from the analysis based on behavioral performance or treatment response. Total subject counts used for each specific analysis are explicitly indicated within the respective figure panels.

### Electrophysiology data analysis

Local field potentials (LFPs) were initially referenced and digitized using 32-bit analog-to-digital conversion. Raw signals were standardized by subtracting the mean and dividing by the standard deviation to ensure unit variance. LFPs were bandpass filtered (1–1000 Hz) and downsampled to 1.5 kHz. Spectral analysis was performed using a multi-taper method with a time-bandwidth product of 4. Following normalization, LFPs were bandpass filtered between 2 and 90 Hz to remove slow drifts and high-frequency noise. A notch filter at 60 Hz was applied to remove line noise. For animals implanted with dual vHPC-targeted LFP wires to more accurately locate the vHPC, a single channel was selected for all subsequent coherence and phase-locking analyses based on two objective criteria. First, priority was given to the channel with the most precise anatomical placement within the vHPC coordinates, as confirmed by post-mortem histology. In instances where both electrodes were correctly positioned, the channel exhibiting the highest signal-to-noise ratio (characterized by minimal line noise and movement artifacts) was selected to ensure the highest fidelity for spectral analysis. Power spectral density and coherence were computed over frequency ranges of 2–90 Hz and 1–90 Hz, respectively.

#### LFP spectrograms

Spectrograms were computed using a 1-s sliding window with 0.5-s steps, employing the short-time Fourier transform. Frequencies above 90 Hz were excluded. The resulting time-frequency power estimates (in dB/Hz) were averaged across trials for each subject, genotype, sex, and treatment group. Condition-specific spectrograms were generated by averaging across trials, and difference maps were computed by subtracting vehicle from BRD group power spectra.

#### vHPC-PFC coherence

Coherence estimates were calculated using the spectral_connectivity Python library, applying a linear detrend to the time series prior to estimation. To minimize the influence of artifacts and noise, an outlier detection step was implemented. Trials were flagged as outliers if their median absolute deviation (MAD)-normalised coherence values exceeded a threshold of 3.3 MAD units (corresponding to 99.9991% of the data) within predefined frequency bands (e.g., 12 to 55 Hz). A maximum of five outlier frequencies were permitted per trial within the 12–55 Hz range. Trials identified as outliers were excluded from subsequent analysis. Coherence values were smoothed using a Gaussian kernel, with the kernel’s standard deviation determined dynamically based on the average spacing of the input frequency bins. Linear interpolation was applied to generate frequency-aligned coherence measures at a standardized set of frequency bins (e.g., 1–90 Hz, in 1 Hz increments). Statistical testing of coherence data followed the same procedure as for behavioural testing based on 3-way ANOVA and FDR-based *p*-value adjustments.

Coherograms were computed using xr-scipy to visualize time-frequency coherence between the vHPC and PFC during both sample and choice runs. LFP signals were downsampled by a factor of 2 to prevent aliasing, bandpass filtered (2–625 Hz), and notch filtered (60, 120, 180 Hz) to remove line noise, followed by normalization. A sliding window of 1 s (750 samples) with 50% overlap was used for coherence computation. Mean coherograms were generated for each group, revealing spectral-temporal patterns of synchronization. Difference maps (BRD3731 minus VEH) were computed to highlight treatment-induced changes. Final heatmaps were averaged across groups.

#### Single unit analysis & phase coupling

Single-unit medial PFC recordings were bandpass filtered at 600–6000 Hz. Spikes were detected using Kilosort V2 [113] and sorted offline and verified by two authors (JP & CA). Only single units with a minimum firing frequency of 0.25 Hz were included in the analysis.

For each identified unit and experimental trial window, spike times were aligned to the analysis window (3 s after door opening). Spike instantaneous phases ${\phi }_{i}$ were extracted from the corresponding Hilbert-transformed vHPC LFP signal, bandpass filtered within the theta frequency range (7–12 Hz).

$${\mathrm{Mean}}\; {\mathrm{Resultant}}\; {\mathrm{Length}}({\mathrm{MRL}}):\,{MRL}=\left|\frac{1}{N}\sum\limits_{i=1}^{N}{e}^{j{\phi}_{i}}\right|$$

$${\mathrm{Pairwise}}\; {\mathrm{Phase}}\; {\mathrm{Consistency}}({\mathrm{PPC}}):{PPC}=\frac{2}{N\left(N-1\right)}\sum\limits_{1\le i < j\le N}\cos \left({\phi}_{i}-{\phi}_{j}\right)$$

MRL reflects the magnitude of the average phase vector of a given unit for N spikes within the analysis window, providing a metric of phase-locking strength. An MRL near 1 indicates strong clustering of spike phases, whereas a value near 0 indicates a more uniform phase distribution. PPC is calculated as the average of the cosine of the phase differences across all pairs of spikes within a given time interval for each unit. This measure quantifies phase-locking while mitigating potential biases from firing rate variations. It is relatively independent of the total spike count and focuses on the consistency of phase relationships across all spike pairs. For statistical consistency, the same outlier trials excluded in the vHPC-PFC coherence analysis were also excluded here. MRL and PPC statistical analysis was based on t-tests between the treated and untreated groups.

## Supplementary information

Supplementary Figures

Supplementary Data 1

Supplementary Data 2

Supplementary Data 3

## Data Availability

The data supporting the current study will become openly available on GitHub at https://github.com/PasseckerLab upon publication. Gene expression data is available on the Gene Expression Omnibus repository upon acceptance.

## Code availability

Custom code supporting the current study will become openly available on GitHub at https://github.com/PasseckerLab upon publication.
