# Supplementary material for: Divergent transcriptomic pathways underlie sex-biased cognitive rescue by developmental GSK3B inhibition in a mouse model of 22q11.2 deletion syndrome
Source: Transl Psychiatry. 2026 Jun 11;16:415. doi: 10.1038/s41398-026-04108-0 (PMC13478295; doi:10.1038/s41398-026-04108-0)
Supplement: Supplementary file 1 [file 41398_2026_4108_MOESM1_ESM.docx]

**Supplementary Figures** - Divergent Transcriptomic Pathways Underlie Sex-Biased Cognitive Rescue by Developmental GSK3B Inhibition in a Mouse Model of 22q11.2 Deletion Syndrome

**Supplementary Figure 1. GSK3A inhibition does not rescue working memory performance in *Df(16)A^+/-^* mice.** (A) Pharmacokinetic validation of paralog-selective GSK3 inhibitors in P7 *Df(16)A^+/-^* brains following intraperitoneal administration of BRD3731 (left) or BRD0705 (right). While Treatment significantly increased drug levels, no significant differences were identified between male and female *Df(16)A^+/-^* mice (Treatment as a factor for BRD3731: p<0.0001 or BRD0705: p=0.0018; Sex for BRD3731: p=0.26 or BRD0705: p=0.64. Interaction: BRD3731: p=0.28 or BRD0705: p=0.51; 2-way ANOVA, n=12 per experiment) (B) Days to reach criterion performance on the spatial working memory task following treatment with the GSK3A inhibitor BRD0705. (Left) Two-way ANOVA (Genotype x Treatment) revealed a significant main effect of Genotype (p=0.0004), indicating that *Df(16)A^+/-^* mice required more days to reach criterion than WT littermates. There was no significant main effect of Treatment (p=0.37), indicating that GSK3A inhibition did not alter performance. (Right) Three-way ANOVA (Sex x Genotype x Treatment) revealed a significant main effect of Sex (p=0.03), with females performing slightly worse overall, and a significant main effect of Genotype (p=0.0002). GSK3A inhibition (BRD0705) had no significant effect on performance in any group. No other main effects or interactions were significant. * p<0.05, ** p<0.01; mice per group indicated within each bar.

***Supplementary Figure 2.*** ***Goal-approach speed is not affected by paralog-selective GSK3 inhibition.*** *(A) Initial training day: Minor speed differences were observed on the first day of training. For the BRD3731 (GSK3B inhibition) experiment (left panel), three-way ANOVA revealed significant main effects of Sex (p<0.0001) and Genotype (p=0.01). For the BRD0705 (GSK3A inhibition) experiment (right panel), three-way ANOVA revealed a significant Sex x Treatment interaction (p=0.001). (B) Final training day: By the last day of training, these initial speed differences were no longer present. In the BRD3731 experiment, no pairwise comparisons revealed significant speed differences. In the BRD0705 experiment, the only significant difference was between vehicle-treated WT and Df(16)A^+/-^ female mice (p=0.008). Crucially, these minor and transient speed differences cannot account for the observed treatment-specific effects on working memory performance. * p<0.05, *** p=0.001, **** p<0.0001; Three-way ANOVA; mice per group indicated within each bar.*

***Supplementary Figure 3.*** ***Data analysis restrictions and outlier removal for coherence analysis.*** *(A) Time window restriction: Analysis of Figure 2 data was restricted to the first three seconds following door opening. This timeframe was chosen to ensure a consistent behavioral epoch across trials, corresponding to the animals' median traversal time of the T-maze stem. (B) Outlier removal: To minimize the influence of artifacts and noise, an outlier detection step was implemented. Trials with median absolute deviation (MAD)-normalised coherence values exceeding 3.3 MAD units within predefined frequency bands were identified as outliers and excluded. The tick mark on the x-axis denotes the cutoff threshold; trials to the left of the mark were included in the analysis. See Methods for further details.*

**Supplementary Figure 4. Quality control and exploratory analysis of PFC transcriptomic data at 1 and 4 weeks.** (A, D) Principal Component Analysis (PCA) plots. PCA plots based on gene expression clustering of P7 (A) and P28 (D) PFC samples after applying RUVr normalization, showing expected subgroup clustering by genotype and sex. (B, E) Verification of 22q11.2 deletion. Log2 fold-change (Log2FC) values from the genotype comparison (*Df(16)A^+/-^* vs. WT) for genes surrounding the 22q11.2 chromosomal region at P7 (B) and P28 (*E*) PFC samples, confirming reduced expression of genes within the deleted region. (C, F) Volcano plots of the Genotype x Sex interaction. Volcano plots showing DEGs for the Genotype x Sex interaction in the P7 (C) and P28 (F) groups. Significant DEGs (adjusted p-value<0.05) are highlighted in red (see Suppl. Data 1 for the full list) and genes significant in both P7 and P28 are orange.

***Supplementary Figure 5. Genotype-dependent differential gene expression by sex*.** Volcano plots illustrate differential expression of genes between *Df(16)A^+/-^* and WT mice. The analysis is stratified by sex (females, left panels; males, right panels) and developmental timepoint (P7, top panels; P28, bottom panels). Genes with significant differential expression (adjusted p<0.05) are highlighted in red. Genes located within the *Df(16)A^+/–^* deletion region are highlighted in green. A complete list of differentially expressed genes is available in Suppl. Data 1.

***Supplementary Figure 6. Cluster-based KEGG pathway enrichment analysis for both developmental time points.*** (*A*) At P7, cluster 1 comprises genes with higher expression in female *Df(16)A^+/-^* mice compared to their male counterparts and shows the greatest number of significantly enriched pathways. In contrast, Cluster 4 represents genes with elevated expression in male *Df(16)A^+/-^* mice relative to male WT mice. Notably, this cluster contained the highest number of pathways associated with *Gsk3b*, including axon guidance, Hippo signaling, Wnt signaling, and signaling pathways regulating pluripotency of stem cells. Cluster 3, which showed an expression pattern similar to Cluster 4 and included *Gsk3b*, was enriched for only a few pathways. (B) At P28, Cluster 1 exhibited an expression pattern reminiscent of Cluster 4 at P7, with increased gene expression in male *Df(16)A^+/-^* mice compared to male WTs. This cluster contained *Gsk3b* and was highly enriched for numerous *Gsk3b*-associated pathways, such as dopaminergic synapse, pathways of neurodegeneration, MAPK signaling, ErbB signaling, mTOR signaling, and insulin resistance. No significant pathway enrichment was identified for Cluster 3 at this timepoint. Only the top 20 KEGG pathways are visualized.

**Supplementary Figure 7: Genotype-driven disruption of sex-specific expression in neurodevelopmental risk genes at P28.** 17 syndromic autism spectrum disorder risk genes (black; SFARI database) and three high-confidence schizophrenia risk genes (red; SCHEMA dataset, q<0.1) were identified among the significant Genotype × Sex interaction DEGs at P28. All those genes present a pattern where the baseline sex difference observed in WT mice (higher expression in females than males) is attenuated or reversed in *Df(16)A^+/-^* mice.
